# An attenuated Zika virus NS4B protein mutant is a potent inducer of antiviral immune responses

**DOI:** 10.1038/s41541-019-0143-3

**Published:** 2019-11-28

**Authors:** Guangyu Li, Awadalkareem Adam, Huanle Luo, Chao Shan, Zengguo Cao, Camila R. Fontes-Garfias, Vanessa V. Sarathy, Cody Teleki, Evandro R. Winkelmann, Yuejin Liang, Jiaren Sun, Nigel Bourne, Alan D. T. Barrett, Pei-Yong Shi, Tian Wang

**Affiliations:** 1 Department of Microbiology and Immunology, University of Texas Medical Branch, Galveston, TX 77555 USA; 2 Department of Biochemistry and Molecular Biology, University of Texas Medical Branch, Galveston, TX 77555 USA; 3 Department of Pathology, University of Texas Medical Branch, Galveston, TX 77555 USA; 4 Sealy Institute for Vaccine Sciences, University of Texas Medical Branch, Galveston, TX 77555 USA; 5 Department of Pediatrics, University of Texas Medical Branch, Galveston, TX 77555 USA; 6 Sealy Center for Structural Biology and Molecular Biophysics, University of Texas Medical Branch, Galveston, TX 77555 USA

**Keywords:** Biotechnology, Immunology, Microbiology, Diseases, Live attenuated vaccines

## Abstract

Live attenuated vaccines (LAVs) are one of the most important strategies to control flavivirus diseases. The flavivirus nonstructural (NS) 4B proteins are a critical component of both the virus replication complex and evasion of host innate immunity. Here we have used site-directed mutagenesis of residues in the highly conserved N-terminal and central hydrophobic regions of Zika virus (ZIKV) NS4B protein to identify candidate attenuating mutations. Three single-site mutants were generated, of which the NS4B-C100S mutant was more attenuated than the other two mutants (NS4B-C100A and NS4B-P36A) in two immunocompromised mouse models of fatal ZIKV disease. The ZIKV NS4B-C100S mutant triggered stronger type 1 interferons and interleukin-6 production, and higher ZIKV-specific CD4^+^ and CD8^+^ T-cell responses, but induced similar titers of neutralization antibodies compared with the parent wild-type ZIKV strain and a previously reported candidate ZIKV LAV with a 10-nucleotide deletion in 3′-UTR (ZIKV-3′UTR-Δ10). Vaccination with ZIKV NS4B-C100S protected mice from subsequent WT ZIKV challenge. Furthermore, either passive immunization with ZIKV NS4B-C100S immune sera or active immunization with ZIKV NS4B-C100S followed by the depletion of T cells affords full protection from lethal WT ZIKV challenge. In summary, our results suggest that the ZIKV NS4B-C100S mutant may serve as a candidate ZIKV LAV due to its attenuated phenotype and high immunogenicity.

## Zika virus vaccination: identifying NS protein mutations for protection

Live attenuated vaccines are used to protect against some flavivirus infections; however, no Zika virus (ZIKV) vaccine has yet been approved for human use. Here, Tian Wang and colleagues use nonstructural protein 4B (NS4B), which is involved in virus replication and evasion of host immunity, as a basis for a potential ZIKV vaccine. Using site-directed mutagenesis, they generated mutations in the conserved N-terminal and central hydrophobic regions of this protein, and identify three single-site mutations that caused the virus to become attenuated. One mutation in particular was found to be more attenuated in an immunocompromised mouse model of ZIKV—the NS4B-C100S mutant. When compared with the wild-type virus strain and a previously mutated ZIKV strain, it induced stronger antiviral immune responses in this mouse model, including increased type I IFN and IL-6 production, and increased ZIKV-specific CD4+ and CD8+ T-cell responses, yet similar neutralizing antibody titerss. Both active and passive immunization with this mutant provided protection when mice were challenged with wild-type ZIKV, suggesting that this mutant strain may warrant further studies as a potential vaccine.

## Introduction

Zika virus (ZIKV), a flavivirus, has caused outbreaks of disease in the Americas and Caribbean in the recent years with more than one million human infections.^1–3^ The virus can be transmitted by mosquito bites, or less frequently by sexual contact.^4–6^ Although the majority of human infections are either asymptomatic or result in a self-limiting flu-like febrile illness, the virus has been associated with severe neurological diseases, such as the autoimmune disorder Guillain-Barre syndrome in adults and congenital Zika syndrome (CZS) in fetuses and infants, including microcephaly, spontaneous abortion, and intrauterine growth restriction.^7,8^ Currently, no vaccines are available for human use. A vaccine to protect against CZS is urgently needed.^9,10^

The single-stranded, positive-sense genome of flaviviruses encodes three structural proteins as follows: capsid, membrane and envelope (E), and seven nonstructural (NS) proteins: NS1, NS2A, NS2B, NS3, NS4A, NS4B, and NS5. The NS4B protein is a 27 KDa, non-glycosylated, hydrophobic homodimer protein. The protein has extensive homology between flaviviruses and is known to be involved in virus replication and evasion of host innate immunity.^11–15^ The two highly conserved coding regions, N-terminal and central hydrophobic of NS4B protein, have been associated with these activities. The N-terminal domain of NS4B protein, ranging from amino acid (aa) 35–aa60 has a resemblance to an immunomodulatory tyrosine inhibitory motif found in various components of mammalian cell-signaling cascades,^16^ which contributes to its antagonist activities for interferon (IFN) and inflammatory cytokine signaling.^12–14^ The central portion of the NS4B protein ranging from aa95 to aa120 has been related to the virulence phenotype of flaviviruses, as mutations made in this region contribute to the attenuated phenotype in Yellow fever virus (YFV), Japanese encephalitis (JE) virus, and dengue virus (DENV). We have previously identified two mutations in wild-type (WT) WNV NY99, one in each of the two domains of NS4B, using site-directed mutagenesis.^17,18^ The WNV NS4B-P38G mutant had significantly reduced neuroinvasiveness^17^ but triggered stronger innate cytokine and memory T-cell responses in mice than did the parent strain WT WNV NY99.^19^ Another mutant, WNV NS4B-C102S, in the central hydrophobic region was highly attenuated for both neuroinvasiveness and neurovirulence in mice.^18^ The NS4B-P38 and NS4B-C102 residues are shared by all mosquito-borne flaviviruses. In this study, we have made several ZIKV NS4B mutants at these two residues and found that the ZIKV NS4B-C100S mutant was the most attenuated one in mice. Moreover, the ZIKV NS4B-C100S mutant induced more potent antiviral innate and adaptive immune responses than the parent WT ZIKV FSS13025 strain and a previously reported candidate live attenuated vaccine (LAV) strain, which provided protection against lethal WT ZIKV challenge.

## Results

### The ZIKV NS4B-C100S mutant is highly attenuated in mice

**Fig. 1:**
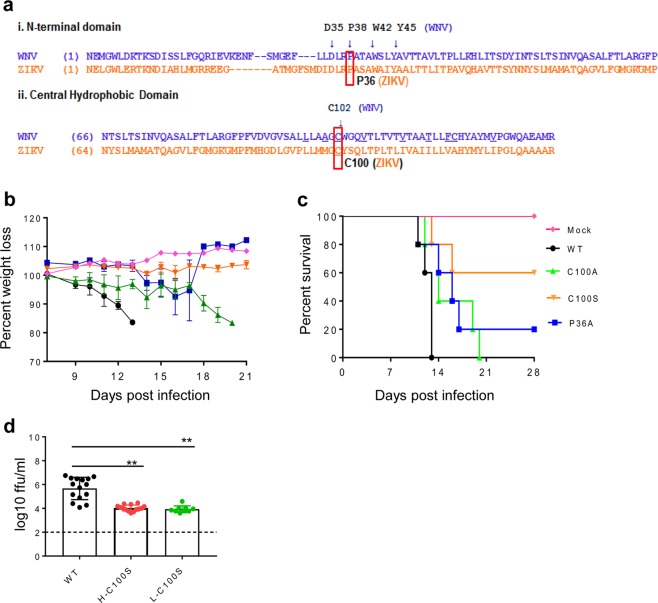
ZIKV NS4B-C100S mutant is attenuated in AG129 mice. **a** Amino acid alignments for WNV and ZIKV in the conserved N-terminal region (i) and central hydrophobic domain (ii) of NS4B. **b**, **c** Six-week-old AG129 mice (*n* = 4 or 5) were immunized i.p. with 10 or 2 × 10^4^ FFU WT and mutant viruses and PBS (mock), respectively. The immunized mice were monitored for weight loss (**b**) and survival (**c**). Weight loss is indicated by percentage, using the weight on the day before immunization to define 100%. **d** Six-week-old AG129 mice (*n* = 4 or 5) were infected with either 10 FFU of WT ZIKV, or a low (L, 2 × 10^4^ FFU) or high (H, 1 × 10^5^ FFU) dose of NS4B-C100S. Viremia were measured on day 3 (*n* = 9 to 15). Data are presented as means ± SEM. **P < 0.01 compared with the WT group (unpaired *t*-test).

We have previously reported that the WNV NS4B-C102S and WNV NS4B-P38G mutants have significantly reduced neuroinvasiveness in mice and conferred protective immunity upon vaccination.^17,19^ The alignment of the NS4B N-terminal and central hydrophobic regions of WNV to ZIKV showed that the ZIKV equivalent residues were P36 and C100 (Fig. 1a). We generated several mutants at these two residues by using site-directed mutagenesis of a ZIKV infectious clone based on strain FSS13025 (FSS13025ic^20^): NS4B-C100S, NS4B-C100A, NS4B-P36A, and NS4B-P36G, which were equivalent to the mutants generated previously for WNV.^17,18^ After transfecting viral RNAs into Vero cells, mutants NS4B-C100S, C100A, and P36A generated increasing numbers of E protein-positive cells (Supplementary Fig. 1a) and >10^6^ PFU/ml infectious viruses (Supplementary Fig. 1b). Sequencing analysis confirmed the NS4B mutations in the recovered viruses. After five rounds of passaging on Vero cells, the engineered mutations were retained (Supplementary Fig. 1c). In contrast, mutant NS4B-P36G RNA generated only a few E protein-positive cells and no infectious virus was recovered from the RNA-transfected cells. Continuous passaging of the NS4B-P36G culture medium for five rounds revealed a revertant virus with P36G-to-P36A change (Supplementary Fig. 1c). The results indicate that the stable ZIKV NS4B-C100S, C100A, and P36A mutants could be produced in cell culture.

To characterize the attenuated phenotype of the other three mutants, mice deficient in IFN-α/β and IFN-γ receptors (AG129) were inoculated intraperitoneally (i.p.) with 1 × 10^4^ focus-forming unit (FFU) of all three mutants. Naive mice (mock) and mice infected with 10 FFU of the parental WT ZIKV FSS13025ic were included as controls. All WT ZIKV FSS13025ic-infected mice developed disease (progressive weight loss and neurologic signs) and required euthanasia within 2 weeks. The ZIKV NS4B-C100A-infected mice showed a delayed lethality (100%) and weight loss compared with WT ZIKV FSS13025ic group. Twenty percent and 60% of ZIKV NS4B-P36A and ZIKV NS4B-C100S-infected groups survived with a mean survival time of 14.5 ± 2.6 days and 14.5 ± 2.1 days, respectively. ZIKV NS4B-P36A-infected mice only showed weight loss before day 14 but remained at normal levels afterwards. On the contrary, none of the NS4B-C100S mutant- infected mice displayed any significant weight loss (Fig. 1b, c and Table 1). In a second study, AG129 mice infected with either a low (2 × 10^4^ FFU) or a high dose (1 × 10^5^ FFU) of ZIKV NS4B-C100S showed significantly reduced viremia levels on day 3 post infection (pi) compared with mice inoculated with 10 FFU of WT ZIKV FSS13025ic (Fig. 1d). Thus, although all three ZIKV NS4B mutants had reduced virulence in AG129 mice, ZIKV NS4B-C100S was more attenuated than the other two mutants.

**Table 1:** Summary of the outcome in AG129 mice infected with WT ZIKV, P36A, C100A, C100S, or PBS (mock).

Group	Number dead (%)	Mean survival time (±SD)	Survivors with weight loss (%)	Total symptoms (%)
WT	5/5 (100%)	12.4 ± 0.9	0/0 (0%)	5/5 (100%)
P36A	4/5 (80%)	14.5 ± 2.6	0/1 (0%)	4/5 (80%)
C100A	5/5 (100%)	15.8 ± 3.5	0/0 (0%)	5/5 (100%)
C100S	2/5 (40%)	14.5 ± 2.1	0/3 (0%)	2/5 (40%)
Mock	0/4 (0%)	NA	0/4 (0%)	0/4 (0%)

Data are representative of two similar experiments

**Fig. 2:**
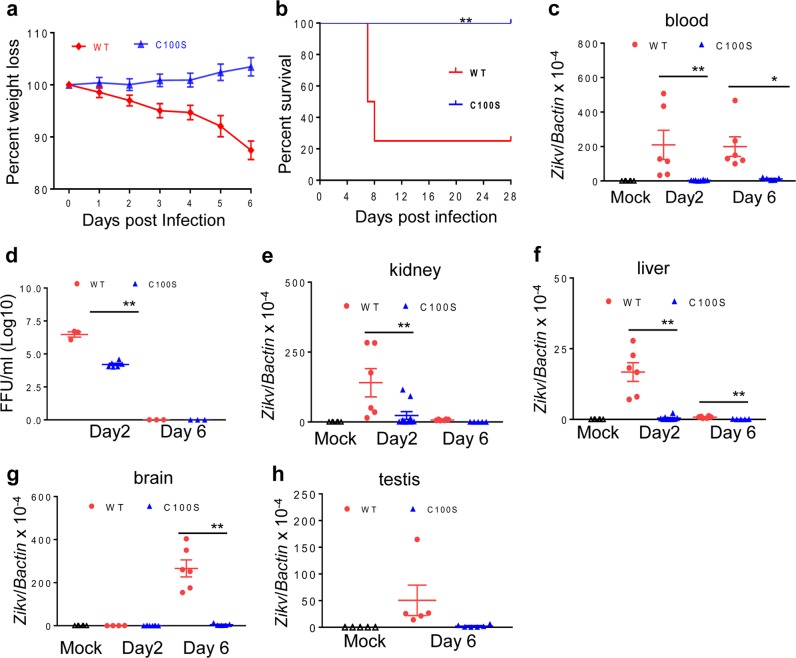
ZIKV NS4B-C100S mutant is attenuated in AB6 mice. **a**, **b** Six-week-old AB6 mice were immunized i.p. with 2 × 10^4^ FFU WT or ZIKV NS4B-C100S. The immunized mice were monitored for weight loss (**a**) and survival (**b**). Weight loss is indicated by percentage, using the weight on the day before immunization to define 100%. *n* *=* 10 and *n* = 8 for WT and NS4B-C100S-infected mice, respectively. **P < 0.01 compared with WT group (log-rank test). **c**–**h** Six-week-old AB6 mice were immunized i.p. with 2 × 10^4^ FFU WT (red dot), ZIKV NS4B-C100S (blue dot), or PBS (mock, open triangle). **c**, **d** Viremia was determined by using Q-PCR and FFA at day 2 and day 6 post infection (pi). **e**–**h** Viral loads in the kidney, liver, brain, and testis were determined by Q-PCR assay. Data are presented as means ± SEM, *n* = 5–10 and are representative two similar experiments. **P < 0.01 or *P < 0.05 compared with the WT group (unpaired *t*-test).

The AG129 mice lack intact IFN-γ signaling and are not ideal for the evaluation of T-cell-mediated adaptive immune responses in the vaccinated mice. We next evaluated the attenuated phenotype of ZIKV NS4B-C100S in mice that are deficient of IFN-α/β receptor (AB6). We inoculated AB6 mice with 2 × 10^4^ FFU of ZIKV NS4B-C100S or the same dose of WT ZIKV FSS13025ic. WT FSS13025ic- infected mice exhibited a significant weight loss within 1 week. In contrast, ZIKV NS4B-C100S-infected mice displayed neither weight loss nor any clinical signs (Fig. 2a). About 75% of AB6 mice succumbed to WT ZIKV FSS13025ic infection, whereas no mortality was observed in the ZIKV NS4B-C100S group (Fig. 2b). The ZIKV NS4B-C100S group had significantly diminished viremia on day 2 pi as determined by focus-forming assay (FFA) and quantitative PCR (Q-PCR; Fig. 2c, d) and 5- to 50-fold lower viral loads detected in the kidney, liver, brain, and testis tissues on days 2 and/or 6 (Fig. 2e–h) pi compared with the WT ZIKV FSS13025ic group. Overall, these results suggest that the ZIKV NS4B-C100S mutant is attenuated in both AG129 and AB6 mouse models of ZIKV infection.

### ZIKV NS4B-C100S induces stronger antiviral innate cytokine and T-cell-mediated immune responses compared with the WT parent strain and a ZIKV-3′UTR-Δ10

**Fig. 3:**
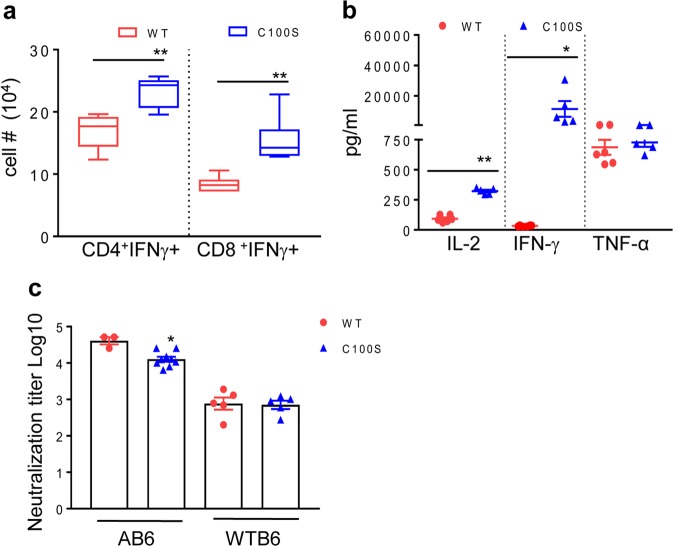
ZIKV NS4B-C100S induces potent antiviral adaptive immune responses. **a**–**c** Six-week-old AB6 (**a**) or WT B6 (**b**) were infected with 2 × 10^4^ FFU WT FSS13025ic (WT) and ZIKV NS4B-C100S (C100S). **a** On day 28, splenocytes of AB6 mice were cultured ex vivo with WT ZIKV for 24 h and were stained for IFN-γ, CD3, and CD4 or CD8. Total number of T-cell subsets per spleen. **b** Supernatants from the ex vivo culture were collected on day 2 after WT ZIKV re-stimulation and measured for Th1-cytokine production. **c** On day 28 after immunization, sera collected from AB6 or B6 mice were measured for NAb titers. Data are presented as means ± SEM, *n* = 3–7 and are representative of two to three similar experiments. **P < 0.01 or *P < 0.05 compared with WT group (unpaired *t*-test).

To determine the immunogenicity of ZIKV NS4B-C100S, AB6 mice were immunized with 2 × 10^4^ FFU of WT ZIKV FSS13025ic and ZIKV NS4B-C100S. On day 28, splenocytes of vaccinated mice were re-stimulated with WT ZIKV antigens in vitro and ZIKV-specific T-cell responses were analyzed using an intracellular cytokine staining method. As shown in Fig. 3a, there were 37–83% more CD4^+^IFN-γ^+^ and CD8^+^IFN-γ^+^ T cells in the ZIKV NS4B-C100S-immunized AB6 mice compared with WT ZIKV FSS13025ic group. Similarly, on day 28, splenocytes of ZIKV NS4B-C100S-vaccinated WT B6 mice produced about 5- to 100-fold higher T-helper (h) 1 type cytokines, such as interleukin (IL)-2 and IFN-γ compared with those of WT ZIKV FSS13025ic-vaccinated WT B6 mice upon ex vivo stimulation with WT ZIKV antigens (Fig. 3b**)**. ZIKV NS4B-C100S-vaccinated WT B6 mice and AB6 mice had similar or slightly lower titers of neutralization antibodies (NAb) in the sera compared with that of WT ZIKV FSS13025ic group, respectively (Fig. 3c). We also measured ZIKV-specific IgM and IgG subtypes in the sera of the vaccinated mice at day 28. As shown in Table 2, ZIKV NS4B-C100S induced similar levels of total IgG, IgG1, and IgG2c responses compared with WT ZIKV FSS13025ic vaccination in both AB6 and WT B6 mice. It appears that ZIKV-specific IgG2c response was the predominant IgG subtype induced in the vaccinated mice. Furthermore, the mutant induced similar and higher IgM titers compared with WT ZIKV FSS13025ic in AB6 and WT B6 mice, respectively.

**Table 2:** Immunoglobulin isotypes in the blood of vaccinated AB6 and WT B6 mice.

Ig isotype	AB6		WT B6	
	WT ZIKV	C100S	WT ZIKV	C100S
IgG	2.24 ± 0.17	2.29 ± 0.12	0.41 ± 0.07	0.57 ± 0.05
IgG1	0.31 ± 0.03	0.33 ± 0.03	0.11 ± 0.00	0.11 ± 0.00
IgG2c	2.64 ± 0.18	2.38 ± 0.14	0.18 ± 0.02	0.14 ± 0.00
IgM	0.42 ± 0.02	0.44 ± 0.03	0.39 ± 0.03	0.70 ± 0.05*

Optical density (OD) of immunoglobulin isotypes and subtypes were determined in sera collected from 4 to 5 vaccinated AB6 or vaccinated WT B6 mice at day 28 after the vaccination. Data are presented as means ± SEM and are representative of at least two similar experiments. *P < 0.05 compared with WT ZIKV-immunized mice (unpaired *t*-test)

**Fig. 4:**
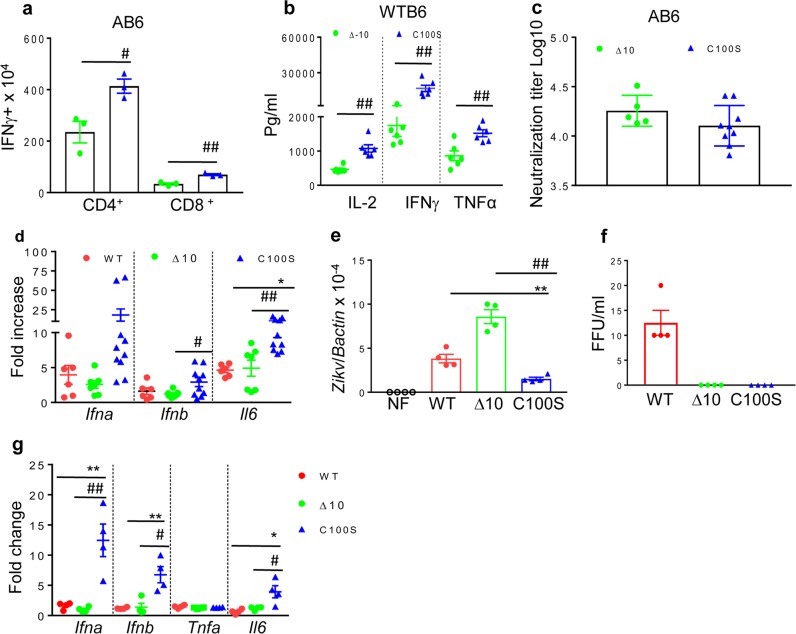
ZIKV NS4B-C100S induces potent antiviral innate and adaptive immune responses compared with ZIKV-3′UTR-Δ10. **a**, **b** Six-week-old AB6 (**a**) mice or WT B6 (**b**) were infected with 2 × 10^4^ FFU ZIKV NS4B-C100S (C100S) or ZIKV-3′UTR-Δ10 (Δ10). **a** On day 28, splenocytes of AB6 mice were cultured ex vivo with WT ZIKV for 24 h and stained for IFN-γ, CD3, and CD4 or CD8. *n* = 3 per group. **b** Supernatants from the ex vivo culture were collected on day 2 after WT ZIKV re-stimulation and measured for Th1- cytokine production. *n* *=* 5 to 7. **c** On day 28 after immunization, mouse sera were measured for NAb titers. **d** Six-week-old AB6 were infected with 2 × 10^4^ FFU WT ZIKV FSS13025ic or ZIKV mutants. On day 2 pi, blood cytokines levels were determined by Q-PCR assay. Data are presented as the fold increase compared with the NF group (*n* = 6 to 10). **e**–**g** AB6 BM macrophages were infected with WT ZIKV FSS13025ic or ZIKV mutants (MOI = 0.1). At day 4 pi, viral load was measured by Q-PCR of viral RNA extracted from infected macrophages (**e**) and FFA (**f**). At D4 pi, cytokine production was determined by using Q-PCR (**g**). Data are presented as the fold increase compared with mock-infected (*n* = 4 per group). Data are presented as means ± SEM and are representative of at least two similar experiments. ^##^*P* < 0.01 or ^#^*P* < 0.05 compared with Δ10 group (unpaired *t*-test). **P < 0.01 or *P < 0.05 compared with the WT group (unpaired *t*-test).

We further compared the immunogenicity of ZIKV NS4B-C100S with another ZIKV LAV candidate ZIKV mutant with 10-nucleotide deletion in the 3′-untranslated region (ZIKV-3′UTR-Δ10), which has been recently reported to be highly attenuated and protect mice from WT ZIKV infection and maternal-to-fetal transmission.^21,22^ To compare the immunogenicity with ZIKV-3′UTR-LAV, 6-week old AB6 or WT B6 mice were vaccinated with 2 × 10^4^ FFU of ZIKV-3′UTR-Δ10 or ZIKV NS4B-C100S. On day 28, there were more than 50% higher CD4^+^IFNγ^+^ and CD8^+^IFNγ^+^ T-cell responses triggered in ZIKV NS4B-C100S-vaccinated AB6 mice compared with those of the ZIKV-3′UTR-Δ10 group. At 1 month post vaccination, splenocytes isolated from ZIKV NS4B-C100S-infected WT B6 mice produced between 0.8- to 8.5-fold more IL-2, IFN-γ, and tumor necrosis factor-α upon stimulation with WT ZIKV antigens compared with those isolated from the ZIKV-3′UTR-Δ10 group (Fig. 4a, b). Interestingly, ZIKV-3′UTR-Δ10 and NS4B-C100S-immunized AB6 mice displayed similar titers of NAbs in sera (Fig. 4c).

To determine the underlying mechanism of immune induction, we next investigated innate cytokine responses in vaccinated mice. As shown in Fig. 4d, there were significantly higher mRNA levels of type 1*Ifn* and *Il6* in the blood of NS4B-C100S-vaccinated mice compared with the other two groups on day 2 pi. Macrophages, which are important antigen-presenting cells (APCs), are permissive to flavivirus infection.^23^ Bone marrow (BM) macrophages of AB6 mice were infected with ZIKV NS4B-C100S and ZIKV-3′UTR-Δ10 at a multiplicity of infection (MOI) of 0.1. On day 4 pi, there were significant lower levels of viral RNA in NS4B-C100S-infected macrophages compared with either WT ZIKV or ZIKV-3′UTR-Δ10 groups. In addition, neither ZIKV-3′UTR-Δ10 nor NS4B-C100S produced detectable viral infectivity titers (Fig. 4e, f). Interestingly, the ZIKV-3′UTR-Δ10-infected cells showed higher viral RNA levels than the WT group, but no detectable viral titer by FFA. This suggests that virus maturation or egress is inhibited in the mutant strain. Furthermore, NS4B-C100S induced about 5- to 7-fold higher mRNA levels of *Ifna*, *Ifnb*, and *Il6* compared with that of either WT ZIKV or ZIKV-3′UTR-Δ10-infected cells (Fig. 4g). Thus, ZIKV NS4B-C100S elicits stronger innate cytokine responses in macrophages, which could lead to more robust T-cell responses in the vaccinated mice. RIG-I-mediated signaling plays an important role in antiviral defense against flavivirus infection.^24^ We also infected BM macrophages isolated from WT B6 mice and mice deficient of MAVS—the adaptor protein for RIG-I (*Mavs*^*−/−*^). Although WT ZIKV and ZIKV-3′UTR-Δ10 produced higher viral loads in *Mavs*^*−/−*^ macrophages, NS4B-C100S remains at the same replication rates in both types of cells (Supplementary Fig. 2a, b). On day 4 pi, ZIKV NS4B-C100S induced similar mRNA levels of *Ifn* and *Il6* in WT and *Mavs*^*−/−*^ macrophages (Supplementary Fig. 2c–e). Overall, these results indicate that RIG-I signaling is dispensable for triggering type 1 IFNs and IL-6, and in control of virus replication following ZIKV NS4B-C100S infection in macrophages.

### Vaccination with the ZIKV NS4B-C100S mutant protects mice from a subsequent WT ZIKV challenge and ZIKV NS4B-C100S-induced humoral immune responses afford full protection from lethal WT ZIKV challenge

**Fig. 5:**
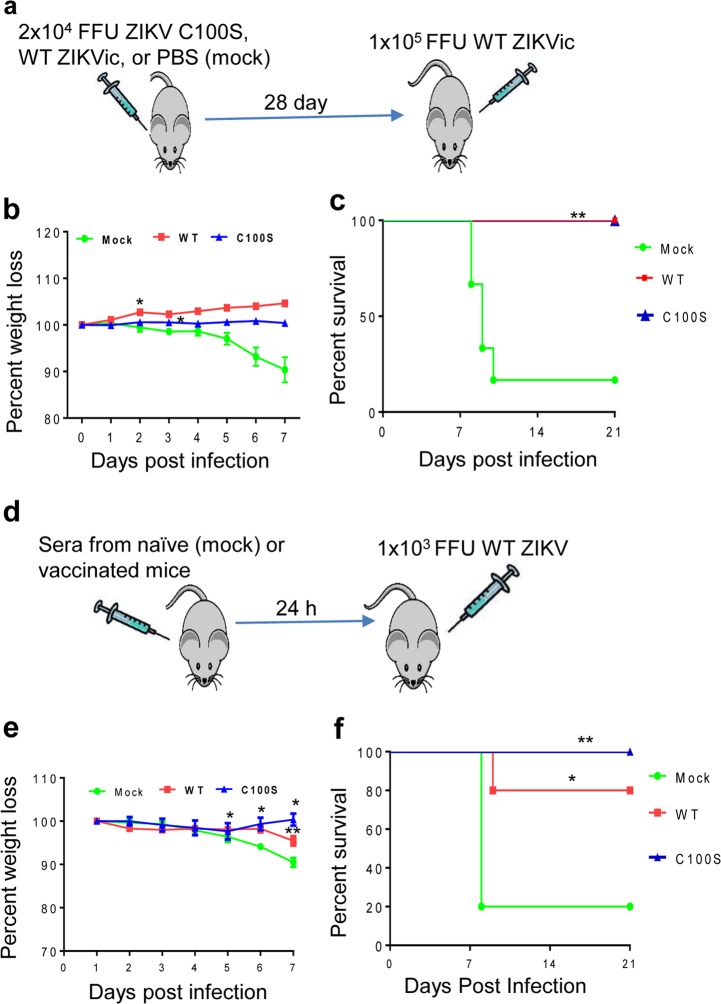
Vaccination with ZIKV NS4B-C100S protects mice from subsequent WT ZIKV challenge. **a**–**c** Six-week-old AB6 mice were infected i.p. with 2 × 10^4^ FFU WT ZIKV FSS13025ic, ZIKV NS4B-C100S, or PBS. On day 28, all surviving mice were challenged with 1 × 10^5^ FFU WT ZIKV FSS13025ic. (**a**) Scheme of active vaccination and challenge. (**b**) Mouse weight loss and (**c**) survival rate. Weight loss is indicated by percentage, using the weight on the day before immunization to define 100%. *n* = 6, 4, and 7 for PBS (mock), WT, and NS4B-C100S-infected mice, respectively. **d**, **f** Six-week-old AB6 mice were injected i.p. with sera collected from naive (mock) or ZIKV WT FSS13025ic, or ZIKV NS4B-C100S-vaccinated mice. All mice were then challenged with 1 × 10^3^ FFU WT ZIKV at 24 h post transfer. (**d**) Scheme of passive vaccination and challenge. (**e**) Mouse weight loss and (**f**) survival rate. Weight loss is indicated by percentage, using the weight on the day before immunization to define 100%. *n* = 5, 5, and 6 for mock sera, WT, and NS4B-C100S-immune sera-transferred mice, respectively. Data are representative of two similar experiments. **c**–**f** **P < 0.01 or *P < 0.05 compared with mock group (log-rank test). **e** **P < 0.01 or *P < 0.05 compared with mock group (unpaired *t*-test).

To determine protective efficacy, 6-week-old AB6 mice were vaccinated with 2 × 10^4^ FFU of ZIKV NS4B-C100S and then challenged with 1 × 10^5^ FFU WT ZIKV FSS13025ic on day 28 pi. Mice immunized with phosphate-buffered saline (PBS) (mock) and the same dose of WT ZIKV FSS13025ic were used as controls (Fig. 5a). Only 25% of WT ZIKV FSS13025ic-immunized mice survived primary infection. During the secondary WT ZIKV challenge, all of the surviving mice vaccinated with either WT ZIKV FSS13025ic or ZIKV NS4B-C100S were protected and showed no clinical signs or weight loss; in contrast, only 25% of the mock group survived the WT ZIKV challenge (Fig. 5b, c). To assess the protective effects of humoral immunity, we adoptively transferred naive 6-week-old AB6 mice with 1:2 dilutions of pooled sera collected from ZIKV NS4B-C100S and WT ZIKV FSS13025ic-vaccinated mice. One day later, all immunized mice were then challenged with 1 × 10^3^ FFU WT ZIKV FSS13025 (Fig. 5d). All mice receiving ZIKV NS4B-100S immune sera survived WT ZIKV challenge, whereas 20% and 80% of the WT ZIKV FSS13025ic immune sera-transferred and the mock groups showed weight loss and succumbed to infection (Fig. 5e, f). Thus, ZIKV NS4B-100S mutant protects mice from WT ZIKV challenge via both active and passive immunizations.

**Fig. 6:**
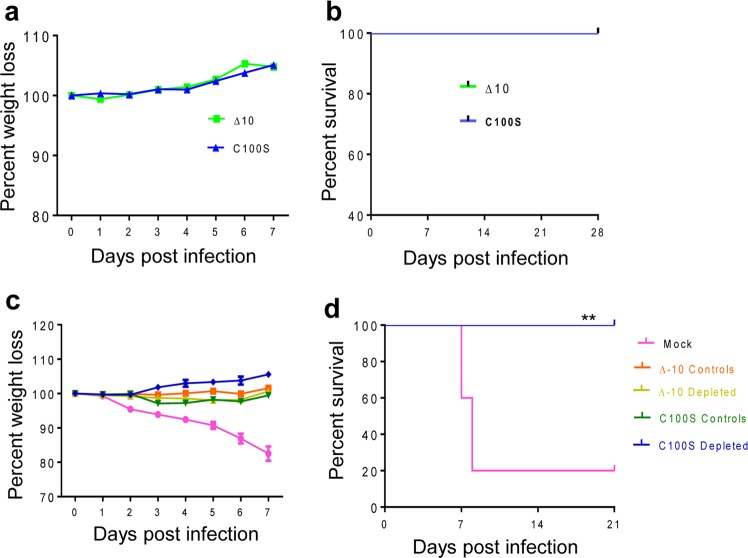
Role of T cells in host protection following vaccination with ZIKV mutants. **a**, **b** Six-week-old AB6 mice were infected i.p. with 2 × 10^4^ FFU ZIKV NS4B-C100S (C100S) or ZIKV-3′UTR-Δ10 (Δ10). (**a**) Mouse weight loss and (**b**) survival rate. Weight loss is indicated by percentage, using the weight on the day before immunization to define 100%. *n* = 10 for each group. **c**, **d** On days 26 and 27, vaccinated mice were either depleted of CD4^+^ and CD8^+^ T cells or injected with PBS followed by challenge with 2 × 10^5^ PFU WT ZIKV FSS13025ic on day 28. (**c**) Mouse weight loss and (**d**) survival rate. Weight loss is indicated by percentage, using the weight on the day before immunization to define 100%. *n* = 5 for each group. **P < 0.01 compared with mock group (log-rank test).

To determine the protective effects of ZIKV LAVs-induced T-cell-mediated immunity upon lethal ZIKV challenge, we inoculated AB6 mice i.p. with 2 × 10^4^ FFU of ZIKV-3′UTR-Δ10 and ZIKV NS4B-C100S. Neither groups showed any weight loss nor clinical signs during a 4-week period following immunization (Fig. 6a, b). The mice were next either depleted of CD4^+^ and CD8^+^ T cells or injected with PBS (controls) 24 h before challenge with 2 × 10^5^ FFU WT ZIKV FSS13025ic. Surprisingly, neither the mice depleted with T cells nor the control non-depleted mice showed any weight loss nor clinical signs. All ZIKV-3′UTR-Δ10 and ZIKV NS4B-C100S-vaccinated mice survived secondary challenge within a 4-week interval. In comparison, 80% PBS-vaccinated AB6 mice (mock group) displayed significant weight loss and succumbed to WT ZIKV infection within 2 weeks (Fig. 6c, d). These results suggest that ZIKV NS4B-C100S-induced humoral immune responses can afford full protection against lethal WT ZIKV challenge.

## Discussion

The introduction of ZIKV to the Americans resulted in a public health emergency and multiple platforms have been utilized in ZIKV vaccine development, including purified inactivated virus, DNA plasmid, mRNA, adenovirus vector, measles virus vector, and LAV. Many of these candidates elicit high levels of neutralizing antibodies and protect mice and/or non-human primates (NHPs) against subsequent ZIKV challenge viremia.^25–28^ Most of the vaccine platforms have focused on the prM and E genes of ZIKV due to a goal of inducing neutralizing antibodies. However, many flavivirus T-cell epitopes have been mapped in NS proteins. The LAV approach is known to have advantages such as using one single dose and induction of durable immunity, and has been successful in controlling flavivirus diseases such as YF and JE. Current candidate ZIKV LAVs have mostly focused on E protein-attenuating mutations or the 3′-UTRs.^22,29,30^ Few studies have focused on the NS protein for vaccine development. Here we showed that the ZIKV NS4B-C100S mutant is highly attenuated in two murine models of ZIKV infection. In addition, we have demonstrated that the ZIKV NS4B-C100S is a potent inducer of innate and adaptive immunity.

T cells play a central role in adaptive immunity during several flavivirus infections.^31–35^ Recent studies in mice and NHPs suggest that both CD4^+^ and CD8^+^ T cells contribute to host protection during ZIKV infection. For example, CD8^+^ T cells are involved in ZIKV clearance.^36,37^ CD4^+^ T cells, in particular their IFN-γ-producing activities, are known to promote humoral immune responses, facilitate virus control, and protect host from ZIKV infection.^38–40^ In this study, we demonstrated that, in both WT B6 and AB6 mice, the ZIKV NS4B-C100S mutant induced more robust Th1-type cellular immune responses than either the parent WT ZIKV or ZIKV-3′UTR-Δ10 mutant. Nevertheless, both passive immunization and T-cell depletion studies suggest that ZIKV NS4B-C100S-induced humoral immunity alone affords full protection against lethal WT ZIKV challenge. Although there was a trend that the passive transfer of serum from C100S-infected animals was more protective than passive transfer from WT virus-infected mice, the latter group showed similar or slightly higher neutralization titers. Overall, the ZIKV NS4B-C100S mutant induces similar levels of protective humoral responses compared with WT ZIKV. In an early study, Larocca et al.^41^ also demonstrated that T cells in mice vaccinated with DNA vaccines expressing ZIKV pre-membrane and envelope (prM-Env) were not involved in protection against WT ZIKV challenge. However, lessons learned from clinical trials of several licensed flavivirus vaccines, including Dengvaxia™ (DENV vaccine) and YFV 17D vaccine, indicate that T-cell immunity is crucial for a safe, efficacious, and durable vaccine.^42,43^ T cells are likely to play a more critical role in induction and/or maintenance of durable immunogenicity. Furthermore, whether T cells are required for the induction of humoral responses remains uncertain. Therefore, future studies will be focused on the role of T-cell immunity in the durability of vaccine immunogenicity and memory B-cell development.

Our results also showed that the ZIKV NS4B-C100S mutant elicited higher type 1 IFNs and IL-6 production compared with WT ZIKV and the ZIKV-3′UTR-Δ10 mutant. Type 1 IFNs and proinflammatory cytokines are known to not only promote APC maturation^44,45^ but also provide a third signal, to synergize with signals from the T cell receptor and co-stimulatory receptors, and optimally activate differentiation and clonal expansion of T cells. For example, IL-6 is known to regulate the expansion and survival of CD4^+^ memory T cells.^46^ In addition, type 1 IFNs are one of the main candidate signal 3 cytokines produced for CD8^+^ T cells in response to intracellular pathogens.^47–51^ Lymphocytoid choriomeningitis virus induces a cytotoxic T-cell response in a type 1 IFN-dependent manner.^52^ These antiviral cytokines triggered by ZIKV NS4B-C100S are likely to contribute to more robust CD4^+^ and CD8^+^ T-cell immune responses in the vaccinated mice. Although MAVS, the adaptor protein of RIG-I receptor, has been shown to be important for host protection against several other flavivirus infections,^24,53^ mice lacking the signaling adapter MAVS exhibited no weight loss, morbidity, or mortality following WT ZIKV infection.^54^ In line with these findings, our data suggest that MAVS is dispensable for triggering type 1 IFNs and IL-6 production, and control of virus replication following ZIKV NS4B-C100S infection in macrophages.

We further performed reporter assays to examine the effects of NS4B and C100S mutation on type 1 IFN production and signaling. The results presented in Supplementary Fig. 3 showed WT NS4B inhibited both type 1 IFN production and signaling. The C100S mutation, however, did not significantly affect the NS4B activity. The results agree with the finding that ZIKV NS4B-C100S triggered similar mRNA levels of *Ifna* and *Ifnb* in *MAVS*^*−/−*^ cells. It is unknown what had contributed to the increased mRNA levels of *Ifna* and *Ifnb* in ZIKV NS4B-C100S-infected animals or cells. Future investigation will be focused on the underlying mechanisms of C100S-triggered higher IFNs and IL-6 production.

Vaccination has proven to be the most effective global strategy for prevention of infectious diseases. Development of a safe and efficacious ZIKV vaccine remains an urgent need. Our results here suggest that the ZIKV NS4B-C100S has important features as a candidate LAV: attenuation and strong immunogenicity. ZIKV NS4B-C100S mutation, alone or in combination with other mutation, will likely confer an attenuated phenotype and the ability to induce at least as high protective immunity as WT ZIKV. Future investigation of the mechanism by which the ZIKV NS mutant induces higher protective immunity can be utilized as a paradigm to aid in the rational development of other efficacious flavivirus LAVs.

## Methods

### Mice

Five to 6-week-old WT C57BL/6 mice (B6), B6 mice deficient of IFN-α/β receptor (AB6), B6 mice deficient of MAVS signaling (*Mavs*^*−/−*^), and 129/Sv mice deficient in IFN-α/β and IFN-γ receptors (AG129) were bred and maintained at the University of Texas Medical Branch (UTMB). Mice were inoculated i.p. with 50 to 1 × 10^4^ FFU of WT ZIKV FSS13025ic and its mutants. In some experiments, mice were re-challenged with 1 to 2 × 10^5^ FFU of WT ZIKV FSS13025ic at day 28 post primary infection. Infected mice were monitored twice daily for signs of morbidity. All animal experiments were approved by the Institutional Animal Care and Use Committee at UTMB.

### Construction of ZIKV NS4B mutants

Standard molecular biology procedures were performed for all plasmid constructions. Mutations were individually introduced into the full length WT ZIKV FSS13025ic by an overlapping PCR method using corresponding primers listed in Table 2. The DNA fragments containing individual mutations were cloned into the pACYC-FLZIKV^20^ through unique restriction sites: NaeI (nucleotide position 6344 of ZIKV genome) and AflII (nucleotide position 8044 of ZIKV genome), and were further verified by DNA sequencing. ZIKV NS4B mutant complementary (cDNA) plasmids were linearized using ClaI enzyme followed by phenol–chloroform purification, and in vitro transcricption using a T7 mMessagemMachine kit (Ambion, Austin, TX). The RNA was precipitated with lithium chloride, washed with 70% ethanol, and re-suspended in RNase-free water. Ten micrograms of RNA transcripts were electroporated into 8 × 10^6^ Vero cells by using Gene Pulser Xcell™ Electroporation Systems (Bio-rad) as per ref. ^20^

### ZIKV infection of cells

BM cells were cultured for 6 days in medium supplemented with macrophage colony-stimulating factor (20 ng/ml, Peprotech), to generate myeloid macrophages.^53^ Cells were infected with ZIKV at a MOI of 0.1 or 2. Culture supernatants and cells were collected at day 4 pi to measure cytokine production.

### Focus-forming assay for viral titer

Vero cell monolayers (American Type Culture Collection) were initially incubated with sample dilutions for 1 h. A semi-solid overlay containing 0.8% methylcellulose (Sigma-Aldrich), 3% fetal bovine serum (FBS), 1% Penicillin–Streptomycin, and 1% l-glutamine (Invitrogen) was then added. At 48 h, the overlay was removed, cell monolayers were washed, air dried, and fixed with 1:1 of acetone:methanol solution for at least 30 min at −20 °C. Cells were next subjected to immunohistochemical staining with either a ZIKV hyperimmune mouse ascitic fluid (World Reference Center for Emerging Viruses and Arboviruses, WRCEVA, Galveston, TX; 1:1000) followed by goat anti-rabbit horseradish peroxidase-conjugated IgG (KPL; 474-1516; 1:1000) at room temperature for 1 h, respectively. After addition of secondary antibody, cells were incubated with a peroxidase substrate (Vector Laboratories, Burlingame, CA) until color developed. The number of foci was counted and used to calculate viral titers expressed as FFU/ml.

### Quantitative PCR

Viral-infected cells or tissues were re-suspended in Trizol (Invitrogen) for RNA extraction. cDNA was synthesized by using a qScript cDNA synthesis kit (Bio-Rad, Hercules, CA). To measure viremia by Q-PCR, RNA was extracted from the blood following red blood cells lysis. The sequences of the primer sets for ZIKV and cytokines cDNA, and PCR reaction conditions were described previously.^55,56^ The PCR assay was performed in the CFX96 real-time PCR system (Bio-Rad). Gene expression was calculated using the formula $2^{-[{\text{C}}_{t} ({\text{target}} \, {\text{gene}})-{\text{C}}_{t}(\beta - actin)]}$.^57^

### Intracellular cytokine staining

The cells isolated from spleen tissues of vaccinated mice and controls were abundant in yield. Splenocytes (2.5 × 10^6^) were incubated with 0.1 × 10^6^ PFU live ZIKV FSS13025 for 24 h. BD GolgiPlug (BD Bioscience) was added to block protein transport at the final 5 h of incubation. Cells were stained with antibodies for CD3 (25-0031-81; 10 µg/ml), CD4 (17-0041-82; 10 µg/ml), or CD8 (11-0081-82, 10 µg/ml), fixed in 2% paraformaldehyde, and permeabilized with 0.5% saponin before adding anti-IFN-γ (12-7311-82; 10 µg/ml) or control rat IgG1 (12-4301-82; 10 µg/ml). All antibodies were purchased from e-Bioscience. Samples were processed with a C6 Flow Cytometer instrument. Dead cells were excluded on the basis of forward and side light scatter. Data were analyzed with a CFlow Plus Flow Cytometer (BD Biosciences).

### Neutralization assay

Mouse sera were first two-fold serially diluted in Dulbecco’s modified Eagle’s medium with 2% FBS and 1% penicillin/streptomycin followed by incubation with mCherry ZIKV at 37 °C for 2 h. The antibody–virus complexes were then added to pre-seeded Vero cells in 96-well plates. At 48 h post infection, cells were visualized by fluorescence microscopy using Cytation 5 Cell Imaging Multi-Mode Reader (Biotek) to quantify the mCherry fluorescence-positive cells. The percentage of fluorescence-positive cells in the non-treatment controls was set to 100%. The fluorescence-positive cells from serum-treated wells were normalized to those of non-treatment controls. A four-parameter sigmoidal (logistic) model in the software GraphPad Prism 7 was used to calculate the neutralization titers (NT_50_).

### Passive immunization

Six-week-old mice were i.p. injected with 200 μl of antiserum (diluted 1 to 2 in PBS) pooled from day 28 WT ZIKV FSS13025ic or ZIKV mutant-infected mice or naive B6 mice. Mice were challenged with 1000 FFU WT ZIKV FSS13025ic 24 h after the serum transfer. Infected mice were monitored twice daily, as described above.

### T-cell depletion

T-cell depletion was achieved by two consecutive injections of 500 µg of anti-CD4 (Bio X Cell; BE0003-1) and anti-CD8 (Bio X Cell; BE0004-1) monoclonal antibodies i.p. at 2 days and 24 h before ZIKV challenge.^41^ Antibody depletions were more than 95% efficient as determined by flow cytometry.

### Cytokine bioplex

Cells (0.3 × 10^6^) were plated in 96-well plates and stimulated with 1.25 × 10^4^ PFU ZIKV FSS13025 for 48 h, supernatants were collected, and cytokine production were measured by using a Bio-Plex Pro Mouse Cytokine Assay (Bio-Rad).

### Statistical analysis

Survival curve comparison was performed using Prism software (GraphPad) statistical analysis, which uses the log-rank test. Values for viral load, cytokine production, and antibody and T-cell response experiments were presented as means ± SEM. *P*-values of these experiments were calculated with a non-paired Student’s *t*-test.

### Reporting summary

Further information on research design is available in the Nature Research Reporting Summary linked to this article.

## Supplementary information

Reporting Summary

Supplementary Information

## Data Availability

All data generated or analyzed during this study are included in this published article (and its Supplementary Information files).
