# Supplementary material for: An attenuated Zika virus NS4B protein mutant is a potent inducer of antiviral immune responses
Source: NPJ Vaccines. 2019 Nov 28;4:48. doi: 10.1038/s41541-019-0143-3 (PMC6883050; doi:10.1038/s41541-019-0143-3)
Supplement: Supplementary file 2 — Supplementary Information [file 41541_2019_143_MOESM2_ESM.pdf]

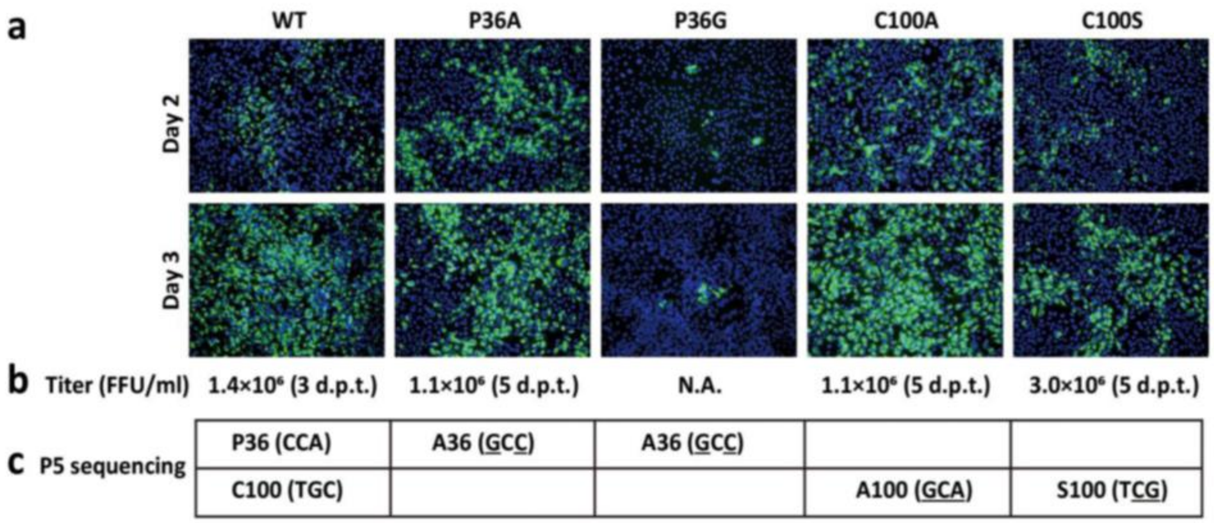

Supplement Figure 1. Characterization of ZIKV NS4B mutants on Vero cells. (**a**) IFA of WT or NS4B mutants on Vero cells. WT or NS4B mutant viral RNAs were transfected into Vero cells. Viral E-positive cells were labeled by 4G2 antibody and indicated in green on day 2 and 3 post-transfection. (**b**) Viral titers for WT or NS4B mutants. The amounts of infectious viruses in culture medium from the transfected cells were quantified by focus-forming assay on Vero cells. The WT and mutant viruses were harvested on day 3 and 5 post-transfection, respectively. No infectious virus could be detected for NS4B-P36G mutant (indicated as N.A.). (**c**) Stability analysis of mutant viruses. The mutant viruses were continuously passaged on Vero cells for five rounds. The passage 5 (P5) viruses were sequenced to examine the stability of the engineered NS4B mutations. The engineered nucleotide changes are underlined.

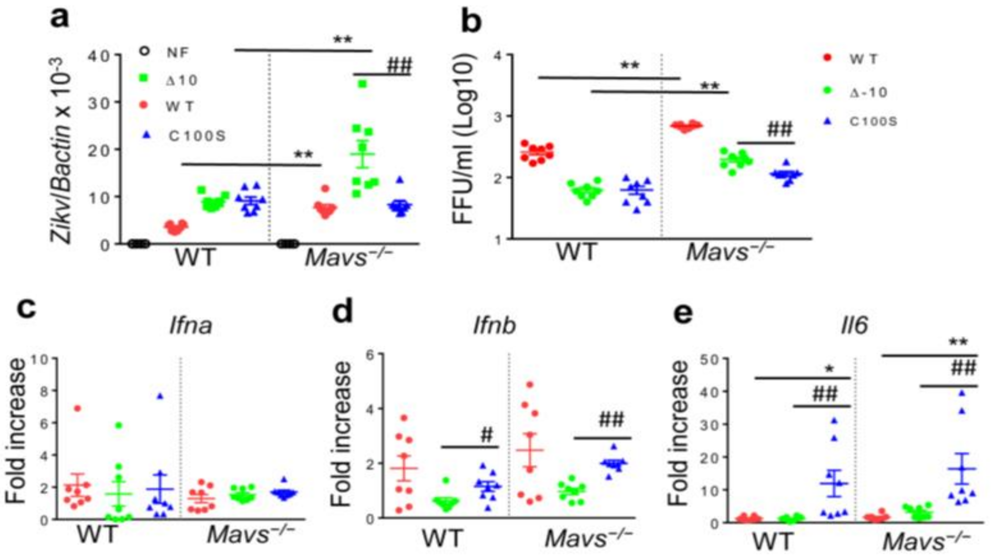

Supplement Figure 2. ZIKV NS4B-C100S induces potent antiviral innate immune responses independent of MAVS signaling. BM-macrophages of WT and *Mavs*$^{-/-}$ mice were infected with ZIKV mutants (MOI =2). At day 4 pi, viral load was measured by Q-PCR (*a*) and FFA (*b*). Cytokine production was determined by using Q-PCR (*c*- *e*). Data are presented as the fold increase compared to mock-infected (n= 4 per group). Data are presented as means ± s.e.m. and are representative of 3 similar experiments. ** $P < 0.01$ or *$P < 0.05$ compared to WT group (Unpaired t test). $^{\#\#}$ $P < 0.01$ or $^{\#}$ $P < 0.05$ compared to Δ10 group (Unpaired t test).

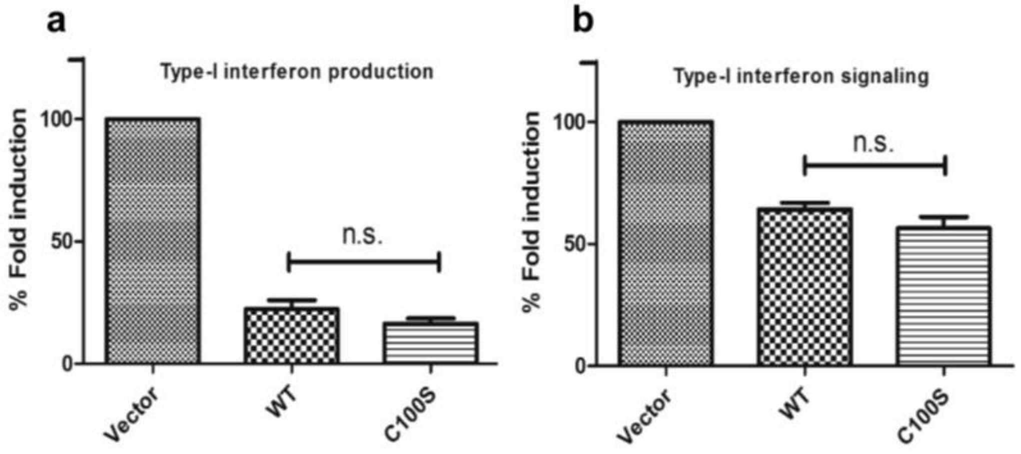

Supplement Figure 3. The effect of NS4B on type 1 IFN production and signaling. For type 1 IFN production, HEK-293T cells were co-transfected with IFN-β promoter firefly luciferase reporters, *renilla* luciferase plasmid, ZIKV NS4B-encoding plasmid or empty control plasmid, and RIG-I-(2CARD) stimulating plasmid. Data were normalized first by *Renilla* luciferase values, and then normalized by none stimulated samples to obtain fold induction. Empty vector samples were set as 100% fold induction. For type-1 IFN signaling, HEK-293T cells were co-transfected with ISRE firefly luciferase reporter plasmid and NS4B expressing plasmid or empty plasmid vector. At 24 h p.i., the cells were treated with 1,000 units/ml of IFN-α for 12 h. The luciferase activities were measured to indicate the IFN-α signaling. Data were presented as mean ± SD. Results are representative of 3 independent experiments with each one in triplicate. No significance (n.s.) indicates $P > 0.05$ compared to WT NS4B (Unpaired t test).

Supplementary Methods:

Indirect immunofluorescence assay (IFA): Transfected cells were seeded in an 8-well Nunc Lab-Tek II Chamber Slide (Thermo Fisher Scientific). At various time points, cells were washed three times with phosphate-buffered saline (PBS) and fixed by cold methanol for 30 min at -20°C. The cells were then washed and incubated in a blocking buffer containing 1% FBS in PBS at room temperature for 1 h. Cells were stained with mouse monoclonal antibody 4G2 (ATCC; HB-112; 1:40), followed with a secondary antibody (Alexa Fluor®488 conjugated goat anti-mouse IgG, Invitrogen, A11001; 1:1000). The cells were washed three times with washing buffer and counterstained with DAPI (4',6-diamidino-2-phenylindole). Images were acquired using an Olympus fluorescence microscope. The images were processed using ImageJ software (National Institutes of Health, MD).

ELISA*:* Microtiter plates were coated with 50 ng/well ZIKV E protein (Bioscience) overnight at 4°C. Sera were diluted 1:40 in PBS containing 0.05% Tween-20 and 8% FBS, and incubated for 1 h at room temperature. Alkaline phosphatase-conjugated goat anti-mouse IgG (Sigma-Aldrich; A3562-5ML; 1:1000), IgG1 (Southern Biotech; 1071-04; 1:1000), $IgG2_C$ (Southern Biotech;1078-04; 1:1000), or IgM (Sigma-Aldrich; A9688-5ML; 1:1000) was then added for 1 h. Color was developed with *p*-nitrophenyl phosphate (Sigma-Aldrich) and intensity read at an absorbance of 405 nm.

Plaque assay: Vero cells (2×10^5 per well) were seeded in 24-well plates 1 day prior to viral infection. Ten-fold serial dilutions of virus samples were prepared in DMEM containing 2% FBS and 1% penicillin/streptomycin and inoculated (100 µl) into each well. After 1 h incubation at 37°C with 5% CO_2, the inoculum was replaced with 500 µl overlay medium. Plaques were developed after incubating the plates at 37°C with 5% CO_2 for 4 days. Following the incubation, methyl cellulose overlay was removed and 0.5 ml methanol-acetone (1:1) solution was added into each well and incubated at room temperature for 15 min. Fixation solution was aspirated and plates were allowed to air dry. Plates were washed three times with PBS and incubated in blocking buffer with 3% FBS, followed by 1 h incubation with ZIKV-specific hyper-immune ascitic fluid (World Reference Center of Emerging Virus and Arboviruses

WRCEVA). Plates were washed three times with PBS followed by 1 h incubation with a secondary antibody conjugated to horseradish peroxidase (Seracare; 5220-0286;1: 2000). Detection of immunostained foci were developed with the addition of aminoethylcarbazole substrate (ENZO Life sciences) according to the vendor's instructions.

Stability studies of NS4B mutant viruses: To examine the stability of NS4B mutants, we passaged the virus on Vero cells for five rounds. Briefly, 1.5×10^{6} Vero cells were seeded into T-25 flask. The virus derived from RNA-transfected cells (defined as P0) was used to infect naive Vero cells. At 5 day post-infection, culture fluid was transferred to a new T-25 flask containing Vero cells in 5 ml of culture medium. After five rounds of such passaging, viral RNAs were extracted from P5 culture fluids using QIAamp Viral RNA Kit (Qiagen) and amplified by RT-PCR using SuperScript III one-step RT-PCR kits (Invitrogen). The RT-PCR products were directed subjected to DNA sequencing.

Type 1 IFN induction and signaling reporter assays: WT and C100S mutant NS4B gene of ZIKV were cloned into expression vector pXJ (1). For the type-1 IFN production assay, HEK-293T cells (ATCC) were co-transfected (1×10^{5} cells per well in 24-well plate) with 5 ng of RIG-I (2CARD) expressing plasmid, 15 ng of IFN-β promoter luciferase reporter plasmid, and 20 ng of NS4B expressing plasmid using X-tremeGENE™ 9 (Roche) with a ratio 1: 2. Empty pXJ vector was used to ensure the same total amount of plasmids in each well. At 36 h, the cells were assayed for dual-luciferase activities per manufacturer's instructions (Promega) For testing type-I IFN signaling, HEK-293T cells were co-transfected with 250 ng of ISRE firefly luciferase reporter plasmid, 20 ng of Rinella luciferase plasmid (as a transfection efficiency control), and 230 ng of NS4B expressing plasmid. At 24 h, 1,000 units/ml of IFN-α (Millipore) was added to the cells. After another 12 h incubation, the cells were assayed for luciferase activities.

Supplementary Table 1. Primers used for the study:

**Primer**	**Sequence (5'-3')**
Zika-6138V	CGACCTGAGGCCGACAAAGTAGCAGC
Zika-8470R	CTTACCACAGCCCGCGTGCCAG
NS4B-P36G-F	GACATTGACCTGCGGggcGCCTCAGCTTGGGCT
NS4B-P36G-R	AGCCCAAGCTGAGGCgccCCGCAGGTCAATGTC
NS4B-P36A-F	GACATTGACCTGCGGgccGCCTCAGCTTGGGCT
NS4B-P36A-R	AGCCCAAGCTGAGGCggcCCGCAGGTCAATGTC
NS4B-C100S-F	CTGCTAATGATAGGTTcgTACTCACAATTAACA
NS4B-C100S-R	TGTTAATTGTGAGTAcgAACCTATCATTAGCAG
NS4B-C100A-F	CTGCTAATGATAGGTgcaTACTCACAATTAACA
NS4B-C100A-R	TGTTAATTGTGAGTAtgcACCTATCATTAGCAG

Supplementary References:

1. Xie, X. *et al.* Inhibition of dengue virus by targeting viral NS4B protein. *J Virol* **85**, 11183-11195, doi:10.1128/JVI.05468-11 (2011).

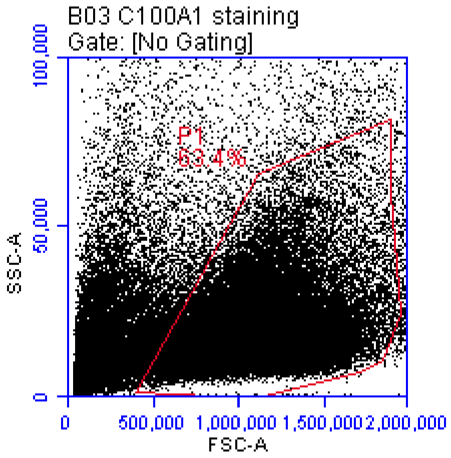
B03 C100A1 staining
Gate: [No Gating]
P1
63.4%
SSC-A
100,000
50,000
0
FSC-A
0
500,000
1,000,000
1,500,000
2,000,000
